# The Impact of Abdominal Fat Levels on All-Cause Mortality Risk in Patients Undergoing Hemodialysis

**DOI:** 10.3390/nu10040480

**Published:** 2018-04-12

**Authors:** Takahiro Yajima, Kumiko Yajima, Hiroshi Takahashi, Keigo Yasuda

**Affiliations:** 1 Department of Nephrology, Matsunami General Hospital, 185-1 Dendai, Kasamatsu, Gifu 501-6062, Japan; 2 Department of Internal Medicine, Matsunami General Hospital, 185-1 Dendai, Kasamatsu, Gifu 501-6062, Japan; green_tea_1324@yahoo.co.jp (Ku.Y.); keigo@matsunami-hsp.or.jp (Ke.Y.); 3 Division of Medical Statistics, Fujita Health University School of Medicine, Aichi 470-1192, Japan; hirotaka@fujita-hu.ac.jp

**Keywords:** hemodialysis, obesity paradox, abdominal fat level, visceral fat area, subcutaneous fat area, all-cause mortality

## Abstract

Although an increased body mass index is associated with lower mortality in patients undergoing hemodialysis (HD), known as the “obesity paradox,” the relationship of abdominal fat levels with all-cause mortality has rarely been studied. We investigated the impact of computed-tomography-measured abdominal fat levels (visceral fat area (VFA) and subcutaneous fat area (SFA)) on all-cause mortality in this population. A total of 201 patients undergoing HD were enrolled and cross-classified by VFA and SFA levels according to each cutoff point, VFA of 78.7 cm^2^ and SFA of 93.2 cm^2^, based on the receiver operator characteristic (ROC) curve as following; group 1 (G1): lower VFA and lower SFA, G2: higher VFA and lower SFA, G3: lower VFA and higher SFA, G4: higher VFA and higher SFA. During a median follow-up of 4.3 years, 67 patients died. Kaplan–Meier analysis revealed 10-year survival rates of 29.0%, 50.0%, 62.6%, and 72.4% in G1, G2, G3, and G4 (*p* < 0.0001), respectively. The adjusted hazard ratio was 0.30 (95% confidence interval [CI] 0.05–1.09, *p* = 0.070) for G2 vs. G1, 0.37 (95% CI 0.18–0.76, *p* = 0.0065) for G3 vs. G1, and 0.21 (95% CI 0.07–0.62, *p* = 0.0035) for G4 vs. G1, respectively. In conclusion, combined SFA and VFA levels were negatively associated with risks for all-cause mortality in patients undergoing HD. These results are a manifestation of the “obesity paradox.”

## 1. Introduction

Obesity is a risk factor for cardiovascular diseases, and it is associated with an increased risk of mortality in the general population [1,2]. Conversely, many studies have reported that an increased body mass index (BMI) is associated with better survival in patients with end-stage renal disease undergoing hemodialysis (HD) [3,4]. This phenomenon has been described as the “obesity paradox.” However, BMI does not discriminate between body fat mass and lean mass, and it poorly reflects body fat distribution [5,6].

Recently, some studies have illustrated that body fat distribution may be more important than overall adiposity; in particular, visceral fat is an independent predictor of dyslipidemia and insulin resistance [7,8] and is associated with metabolic risk factors [9]. Some studies reported that the abdominal visceral fat area (VFA), but not the subcutaneous fat area (SFA), was associated with all-cause mortality in the general population, but the results were controversial [10,11,12,13,14,15,16]. In patients undergoing HD, a study suggested that increased VFA is a risk factor for cardiovascular death [17]. Thus, the association between abdominal fat levels and all-cause mortality remains unclear in this population.

The aim of this study was to investigate the impact of CT-measured abdominal fat level, including VFA and SFA, on all-cause mortality in patients undergoing HD.

## 2. Materials and Methods

### 2.1. Study Participants

Two hundred and one patients undergoing HD who underwent abdominal CT for malignancy screening as part of an annual examination at the outpatient clinic of Matsunami General Hospital were enrolled between January 2005 and December 2015. Patients with advanced malignancy were excluded. This study adhered to the principles of the Declaration of Helsinki, and the study protocol was approved by the ethics committee of Matsunami General Hospital (No. 373).

### 2.2. Data Collection

The following patient information was collected from medical records: age; gender; duration of HD; previous history of smoking, diabetes, hypertension, and cardiovascular disease (CVD); dry weight; and height. In this study, CVD included heart failure, angina pectoris, myocardial infarction, and stroke. Blood samples were obtained from patients in the supine position before the initiation of the HD session, and laboratory data obtained during the same month when CT was performed were used. VFA and SFA were determined using CT, which was performed after an HD session using a 16-slice multi-detector CT scanner (Siemens SOMATOM^®^ Sensation Cardiac 16-slice scanner; Siemens Medical Solutions USA Inc., Malvern, PA, USA), and 10-mm-thick slices were acquired. VFA was defined as the intra-peritoneal fat area, and SFA was defined as the extra-peritoneal fat area between the skin and muscle at the umbilical level. The analysis was conducted using commercially available software (Fat Checker, VOX-BASE; J-MAC system, Sapporo, Japan). In this study, BMI was calculated from the dry weight and height as follows: dry weight (kg)/height^2^ (m^2^).

### 2.3. Follow-Up Study

The study endpoint was all-cause mortality. The cutoff points of VFA and SFA, which predict all-cause mortality, were determined with the use of receiver operator characteristic (ROC) curve respectively. Patients were divided into four groups according to each cutoff point. Patients were followed up for as many as 10 years.

### 2.4. Statistical Analyses

Normally distributed variables are expressed as the mean ± standard deviation, and non-normally distributed variables are given as the median and interquartile range. ROC curves were used to determine cutoff points of VFA and SFA, which predict all-cause mortality. Patients were divided into four groups according to each cutoff point. Regarding comparisons of differences among the groups, continuous variables were analyzed via one-way analysis of variance or the Kruskal–Wallis test, whereas the chi-squared test was utilized for categorical variables. To determine the factors correlated with VFA or SFA, multivariate regression analysis was used. The Kaplan–Meier method was used to estimate survival, which was analyzed using the log-rank test. Hazard ratios (HRs) and 95% confidence intervals (CIs) for all-cause mortality were assessed using Cox proportional hazard regression analysis. The multiple regression model included covariates that were significant at *p* < 0.05 in the univariate analysis.

To assess whether the accuracy of predicting mortality would improve after the addition of the abdominal fat level to a baseline model with covariates significant at *p* < 0.05 in the univariate analysis, the C-index, net reclassification improvement (NRI), and integrated discrimination improvement (IDI) were calculated. The C-index was defined as the area under the ROC curves between individual predictive probabilities for mortality and the incidence of mortality, and it was compared between the baseline model and the model including the abdominal fat level [18]. The NRI is a relative indicator of the number of patients for whom the predicted probabilities for mortality improve, whereas IDI represents the average improvement in predicted probabilities for mortality after adding variables to the baseline model [19].

All statistical analyses were performed using the SPSS version 21 software program (IBM Corp., Armonk, NY, USA). *p* Values of <0.05 were considered to be statistically significant.

## 3. Results

### 3.1. Baseline Characteristics

The baseline characteristics of the study patients are shown in Table 1. The median duration of HD was 1.9 (0.4–27.2) months. The mean BMI, VFA, and SFA were 21.3 ± 3.4 kg/m^2^, 66.4 ± 49.5 cm^2^, and 112.1 ± 64.3 cm^2^, respectively. Multivariate regression analysis revealed that VFA was independently correlated with diabetes mellitus (β = 0.115, *p* = 0.031) and BMI (β = 0.631, *p* < 0.0001). SFA was independently correlated with male gender (β = −0.292, *p* < 0.0001), diabetes mellitus (β = 0.117, *p* = 0.0065), and BMI (β = 0.725, *p* < 0.0001) (Table 2).

### 3.2. Abdominal Fat Level and Mortality

**Figure 1:**
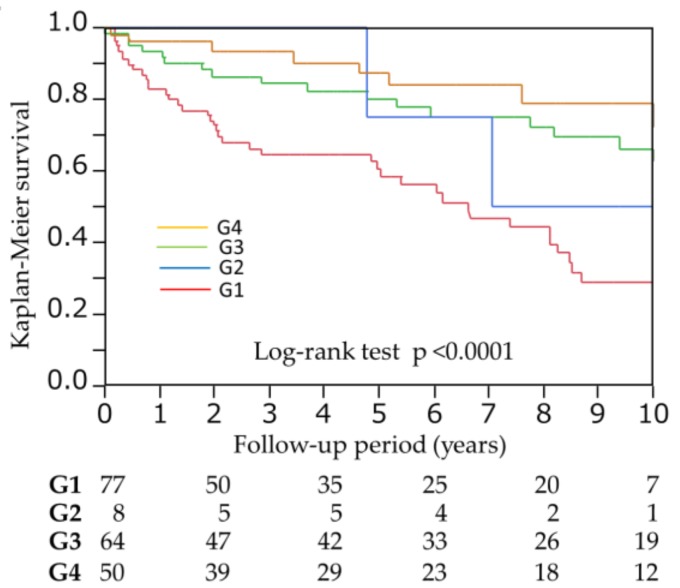
Meier survival curves for all-cause mortality among divided four groups according to each cutoff point of VFA and SFA, which predict mortality. G1: VFA < 78.7 cm^2^, SFA < 93.2 cm^2^; G2: VFA ≥ 78.7 cm^2^, SFA < 93.2 cm^2^; G3: VFA < 78.7 cm^2^, SFA ≥ 93.2 cm^2^; G4: VFA ≥ 78.7 cm^2^, SFA ≥ 93.2 cm^2^. VFA, visceral fat area; SFA, subcutaneous fat area.

During the follow-up period (median, 4.3 [1.4–8.6] years), 67 patients died (infection, 26 [38.8%]; CVD, 23 [34.3%]; malignancy, seven (10.4%); and others, 11 [16.4%]). In univariate Cox proportional hazards analysis, both VFA and SFA were significant predictors for all-cause mortality (HR 0.992, 95%CI 0.985–0.998, *p* = 0.0063; HR 0.991, 95%CI 0.986–0.995, *p* < 0.0001). ROC analysis revealed that the cutoff points of VFA and SFA, which predict all-cause mortality, were 78.7 cm^2^ (AUC 0.61) and 93.2 cm^2^ (AUC 0.67), respectively. Patients were divided into four groups according to each cutoff point (group 1 (G1): VFA < 78.7 cm^2^, SFA < 93.2 cm^2^; G2: VFA ≥ 78.7 cm^2^, SFA < 93.2 cm^2^; G3: VFA < 78.7 cm^2^, SFA ≥ 93.2cm^2^; G4: VFA ≥ 78.7 cm^2^, SFA ≥ 93.2 cm^2^). After 10 years of follow-up, the Kaplan–Meier survival rates were 29.0%, 50.0%, 62.6%, and 72.4% in G1, G2, G3, and G4 (*p* < 0.0001), respectively (Figure 1). After adjusting for age, male gender, previous history of CVD, BMI, total cholesterol, creatinine, albumin, phosphorus, and C-reactive protein, which were significant at *p* < 0.05 in the univariate analysis, adjusted HR was 0.30 (95% confidence interval [CI] 0.05–1.09, *p* = 0.070) for G2 vs. G1, 0.37 (95% CI 0.18–0.76, *p* = 0.0065) for G3 vs. G1, and HR 0.21 (95% CI 0.07–0.62, *p* = 0.0035) for G4 vs. G1, respectively (Table 3).

As for model discrimination, the C-index was greater in the model including cross-classified with VFA and SFA than in the baseline model, but did not reach at significance (0.798 vs. 0.817, *p* = 0.075). The NRI and IDI were significantly improved after adding to the baseline model (NRI = 0.612, *p* = 0.00002; IDI = 0.066, *p* = 0.00005, respectively) (Table 4).

The interaction between VFA and SFA levels for all-cause mortality was not found (*p* = 0.37).

## 4. Discussion

In the present study, higher VFA and lower SFA tended to be associated with a reduced risk for all-cause mortality in patients undergoing HD. In addition, lower VFA and higher SFA or higher VFA and higher SFA was independently associated with a reduced risk for all-cause mortality even after adjusting for other confounders. Moreover, the accuracy of predicting all-cause mortality improved after the addition of cross-classification with VFA and SFA to a predictive model with established risk factors, including BMI. It seems likely that these results are evidence of the “obesity paradox” in this population.

In patients undergoing HD, the concept of “obesity paradox”, in which a higher BMI is paradoxically associated with better survival, is well known [3,4]. Possible explanations for this paradox include protein-energy wasting (PEW), defined as a loss of body protein mass and fuel reserves (fat masses) [20,21]. PEW can be induced by the increased release or activation of inflammatory cytokines such as IL-6 or TNF-α [22]. During inflammatory conditions or malnutrition, body protein stores are diverted to defend against inflammation and repair injury. Thus, the increased body mass of overweight patients undergoing HD provides protection against or resources for responding to inflammation, infection, and subsequent CVD [23]. Therefore, obesity may potentially attenuate the magnitude of PEW and/or inflammation, which would be favorable to patients undergoing HD.

However, BMI, which is widely used as a measure of body composition, does not consider the distribution of body fat, nor can it distinguish body fat mass from lean body mass. Several recent studies have suggested that body fat distribution may be more important than overall adiposity; specifically, visceral fat is related to a greater risk of metabolic diseases, but subcutaneous fat is associated with beneficial metabolic effects [24,25]. Meanwhile, the association between abdominal fat as measured by CT and mortality has been recently investigated [10,11,12,13,14,15,16,17]. In the general population, many previous studies reported that increased VFA is a strong risk factor for all-cause mortality [10,11,12,15], but conflicting results have been reported [13,14,16]. In patients undergoing HD, only a study suggested that increased VFA was a risk factor for cardiovascular mortality (the cutoff determined using ROC curves was 71.5 cm^2^), but all-cause mortality was similar between the low and high VFA groups (38.8% vs. 35.1%). Therefore, the association between abdominal fat levels and all-cause mortality remains controversial in patients undergoing HD.

In this study, higher VFA and lower SFA tended to be associated with reduced risks for all-cause mortality after the adjustment (*p* = 0.070), but patients with higher VFA and lower SFA were very small. Thus the impact of higher VFA and lower SFA for mortality might have been not enough to evaluate. However, patients with large visceral adipose tissue and small subcutaneous adipose tissue might be generally rare. The results of our study were opposite to that of previously reported findings, but only a study of an “elderly” Asian population illustrated that increased VFA was associated with better survival [14]. East Asian populations, including Japanese subjects, exhibit the largest accumulation of visceral adipose tissues but the lowest accumulation of subcutaneous adipose tissues despite lower absolute BMI levels; therefore, they have the most deleterious abdominal fat distribution [26]. On the other hand, visceral adipose tissues secrete higher levels of inflammatory cytokines than subcutaneous adipose tissues [27], and it is associated with insulin resistance and markers of oxidative stress and inflammation [28,29], which may predict malnutrition and the development of PEW [30,31]. Thus, the VFA levels in our participants may have been too low to increase the risk of all-cause mortality.

Conversely, lower VFA and higher SFA was significantly associated with a reduced risk of all-cause mortality. The effect of SFA on all-cause mortality is controversial in the general population, but it has been recently reported that high SFA is associated with a reduced mortality risk in patients with cancer [32]. In obese patients with cancer, excess adipose tissues may provide fuel to bridge the gap between decreased energy intake and elevated energy requirements. In this study, possible reasons for the survival benefit of increased SFA may be explained as follows. First, SFA, as energy storage, may reflect the overall nutritional status, as SFA was significantly correlated with BMI. Second, increased subcutaneous adipose tissues may have beneficial metabolic effects, such as protection against insulin resistance [25], as observed in the general population. In patients undergoing HD, who have a short life expectancy, the long-term effects of obesity as a conventional risk factor for mortality may be overwhelmed by the short-term effects of undernutrition.

Interestingly, higher VFA and higher SFA were significantly associated with reduced risks for mortality. The precise mechanisms why increased VFA and SFA were protective for survival in HD patients remain unknown, but these results may be exactly “obesity paradox”. In addition, the accuracy of predicting all-cause mortality improved after the addition of cross-classification with VFA and SFA into a predictive model with established risk factors, including BMI; therefore, decreased abdominal fat levels may be a strong surrogate marker of PEW in patients undergoing HD.

There were several limitations to this study. First, the number of enrolled patients in this retrospective single-center study was relatively small. Second, the duration of HD at the time of CT varied. Third, muscle mass, another important component of PEW, was not evaluated. In addition, changes of abdominal fat levels during the long-term follow-up period were not considered. Further large studies may be needed to validate our results.

## 5. Conclusions

Combined SFA and VFA levels were negatively associated with risks for all-cause mortality in patients undergoing HD. These results may be an example of the “obesity paradox.”

## Figures and Tables

**Table 1:** Baseline patient characteristics.

	All Patients (*N* = 201)	G1 (*N* = 79)	G2 (*N* = 8)	G3 (*N* = 64)	G4 (*N* = 50)	*p* Value
Age (years)	63.3 ± 13.1	64.9 ± 12.1	68.9 ± 9.9	60.6 ± 15.4	63.3 ± 11.2	0.14
Male (%)	72.1	77.2	87.5	60.9	76.0	0.10
Duration of HD (months)	1.9 (0.4–27.2)	2.3 (0.4–23.4)	1.9 (0.3–5.3)	1.9 (0.5–28.4)	1.2 (0.4–29.4)	0.57
Diabetes (%)	45.2	29.1	50.0	56.3	56.0	0.0027
Hypertension (%)	98.0	98.7	100	96.9	98.0	0.82
Smoking (%)	23.9	20.3	0	23.4	34.0	0.056
Previous CVD (%)	76.6	72.2	87.5	79.7	78.0	0.60
BMI (kg/m^2^)	21.3 ± 3.4	18.7 ± 1.9	21.2 ± 1.8	21.9 ± 2.5	24.7 ± 3.2	<0.0001
BUN (mg/dL)	56.0 ± 14.7	54.3 ± 16.1	52.5 ± 19.7	56.4 ± 12.6	58.6 ± 14.2	0.39
Creatinine (mg/dL)	8.6 ± 3.0	8.26 ± 2.99	9.4 ± 4.0	8.6 ± 3.0	9.0 ± 3.0	0.51
Albumin (g/dL)	3.7 ± 0.4	3.7 ± 0.5	3.6 ± 0.5	3.8 ± 0.4	3.7 ± 0.3	0.55
Hemoglobin (g/dL)	10.6 ± 1.5	10.5 ± 1.5	10.3 ± 1.8	10.7 ± 1.6	10.6 ± 1.4	0.76
T-Cho (mg/dL)	153 ± 34	151 ± 33	149 ± 38	150 ± 30	161 ± 40	0.32
Uric acid (mg/dL)	7.0 ± 1.5	6.6 ± 1.4	6.4 ± 2.4	7.1 ± 1.5	7.4 ± 1.5	0.020
Ca (mg/dL)	8.9 ± 0.9	8.9 ± 1.0	9.3 ± 1.0	8.9 ± 0.8	8.9 ± 1.0	0.76
P (mg/dL)	5.2 ± 1.4	4.8 ± 1.2	4.7 ± 1.3	5.4 ± 1.3	5.7 ± 1.6	0.0014
Glucose (mg/dL)	146 ± 64	131 ± 48	192 ± 103	146 ± 64	160 ± 73	0.013
CRP (mg/dL)	0.14 (0.06–0.50)	0.12 (0.05–0.53)	0.13 (0.07–0.42)	0.11 (0.04–0.36)	0.29 (0.13–0.72)	0.71
VFA (cm^2^)	66.4 ± 49.5	32.0 ± 19.0	107.2 ± 21.4	50.3 ± 17.0	135 ± 41.6	<0.0001
SFA (cm^2^)	112.1 ± 64.3	59.0 ± 22.7	80.3 ± 6.3	138.9 ± 52.3	166.8 ± 61.7	<0.0001

HD, hemodialysis; CVD, cardiovascular disease; BMI, body mass index; BUN, blood urea nitrogen; CRP, C-reactive protein; T-Cho, total cholesterol; VFA, visceral fat area; SFA, subcutaneous fat area. G1: VFA < 78.7 cm^2^, SFA < 93.2 cm^2^; G2: VFA ≥ 78.7cm^2^, SFA < 93.2 cm^2^; G3: VFA < 78.7 cm^2^, SFA ≥ 93.2 cm^2^; G4: VFA ≥ 78.7 cm^2^, SFA ≥ 93.2 cm^2^.

**Table 2:** Relationships between abdominal fat levels and baseline variables according to multivariate regression analysis.

	VFA		SFA	
Variables	β	*p* Value	β	*p* Value
Age	-	-	−0.080	0.075
Male gender	-	-	−0.292	<0.0001
Diabetes	0.115	0.031	0.117	0.0065
Smoking	0.018	0.74	-	-
BMI	0.631	<0.0001	0.725	<0.0001
Phosphorus	0.083	0.12	0.048	0.28

VFA, visceral fat area; SFA, subcutaneous fat area.

**Table 3:** Cox proportional hazards analysis of the risk of all-cause mortality in patients undergoing hemodialysis.

	Univariate		Multivariate	
	HR (95% CI)	*p* Value	HR (95% CI)	*p* Value
cross-classified with VFA and SFA		<0.0001		0.012
G2 (vs. G1)	0.46 (0.08–1.51)	0.23	0.30 (0.05–1.09)	0.070
G3 (vs. G1)	0.37 (0.21–0.66)	0.0005	0.37 (0.18–0.76)	0.0065
G4 (vs. G1)	0.24 (0.10–0.49)	<0.0001	0.22 (0.07–0.62)	0.0035

VFA, visceral fat area; SFA, subcutaneous fat area; HR, hazard ratio; CI, confidence interval. The multivariate model included all variables significant at *p* < 0.05 in the univariate analysis (age, male gender, BMI, previous history of cardiovascular disease, creatinine, total cholesterol, albumin, phosphorus, and C-reactive protein). G1: VFA < 78.7 cm^2^, SFA < 93.2 cm^2^; G2: VFA ≥ 78.7 cm^2^, SFA < 93.2 cm^2^; G3: VFA < 78.7 cm^2^, SFA ≥ 93.2 cm^2^; G4: VFA ≥ 78.7 cm^2^, SFA ≥ 93.2 cm^2^.

**Table 4:** Predictive value of the abdominal fat levels for all-cause mortality using the C-index, net reclassification improvement (NRI), and integrated discrimination improvement (IDI).

Variable	C-Index	*p* Value	NRI	*p* Value	IDI	*p* Value
Established risk factors	0.784 (0.716–0.853)	Reference	Reference		Reference	
+cross-classified with VFA and SFA	0.817 (0.753–0.881)	0.075	0.612	0.00002	0.066	0.00005

Established risk factors: age, male gender, BMI, previous history of cardiovascular disease, creatinine, total cholesterol, albumin, phosphorus, and C-reactive protein.
